# B-Cell Translocation Gene 2 Upregulation Is Associated with Favorable Prognosis in Lung Adenocarcinoma and Prolonged Patient Survival

**DOI:** 10.1155/2022/1892459

**Published:** 2022-09-14

**Authors:** Junting Liang, Linna Cheng, Haiyan Gao, Wanying Fu, Xiulei Zhang, Jianhua Zhang, Jinming Li, Chuanliang Chen

**Affiliations:** ^1^Clinical Bioinformatics Experimental Center, Henan Provincial People's Hospital, People's Hospital of Zhengzhou University, Zhengzhou, 450003 Henan, China; ^2^Institute of Hematology, Henan Key Laboratory of Stem Cell Differentiation and Modification, Henan Provincial People's Hospital, Zhengzhou University People's Hospital, Henan University People's Hospital, Zhengzhou, 450003 Henan, China; ^3^Henan Key Laboratory of Neurological Imaging, Henan Provincial People's Hospital, People's Hospital of Zhengzhou University, Zhengzhou University, Zhengzhou, 450003 Henan, China; ^4^School of Basic Medicine, Xinxiang Medical University, Xinxiang, 453003 Henan, China; ^5^Department of Microbiome Laboratory, Henan Provincial People's Hospital, People's Hospital of Zhengzhou University, Zhengzhou, 450003 Henan, China; ^6^Medical Engineering Technology and Data Mining Institute of Zhengzhou University, Zhengzhou, 450000 Henan, China; ^7^The Translational Research Institute, Henan Provincial People's Hospital, School of Medicine, Zhengzhou University, Zhengzhou, 450003 Henan, China

## Abstract

The tumor suppressor protein B-cell translocation gene 2 (BTG2) is downexpressed in lung adenocarcinoma (LUAD); however, its role in LUAD survival remains unknown. This investigation is aimed at exploring the activity of BTG2 in LUAD. We analyzed BTG2 expression in LUAD datasets of the TCGA database and examined that BTG2 was markedly downregulated in comparison with adjacent normal tissues. The prognostic analysis suggested that higher expression of BTG2 protein correlates with prolonged survival in patients. Vectors expressing BTG2 were stably transduced into lung adenocarcinoma A549 cells. The overexpression of BTG2 in A549 cells causes cellular G1 phase arrest but did not affect cell proliferation, accompanied by increased activation of NF-*κ*B. Our data indicate that BTG2 overexpression may trigger an autoregulatory prosurvival NF-*κ*B pathway, which is resistant to environmental intervention owing to an increased level of BTG2.

## 1. Introduction

Among the most frequently occurring cancers is that associated with the lungs. It is one of the major causes of mortality worldwide [1, 2]. Lung adenocarcinoma (LUAD) is one of the most frequently occurring non-small-cell cancer [3]. Early disease diagnosis and new treatment methods need further investigation to determine the underlying molecular mechanisms responsible for the incidence and development of LUAD.

Tumor suppressor genes (TSGs) generally regulate cell cycle arrest, apoptosis, or protein ubiquitination [4–9]. Despite the importance of TSGs in tumorigenesis, the underlying molecular mechanisms of TSGs in lung cancer progression are still unclear. Therefore, more studies are required to understand the roles of TSGs in LUAD.

B-cell translocation gene-2 (BTG2) is a BTG/TOB gene family member [10]. Studies have shown that overexpression of BTG2 can repress in vitro cellular growth [11] and BTG2 serves as a tumor suppressor gene in various types of malignant tumors [12–14]. Previous reports suggest that BTG2 is downregulated in various malignant tumors, including prostate cancer [15], lung cancer [16], and hepatic cell carcinoma [17]. It has been discovered that the downregulation of BTG2 is linked with substandard breast carcinoma prognosis [18, 19]. Many investigations have indicated that BTG2 overexpression could stimulate cellular apoptosis and restrain cell invasion in prostate cancer [20], medulloblastoma [21], and breast cancer [22]. However, the underlying molecular mechanisms of BTG2 in LUAD remained unclear.

The current study is aimed at using the TCGA database to locate the gene BTG2 as a downregulated gene and predict prognosis and survival in LUAD patients, developing the human lung adenocarcinoma A549 cell line with BTG2 overexpression, and investigating its function experiments to assess its role in tumor progression.

## 2. Materials and Methods

### 2.1. Identification of Differentially Expressed Genes (DEGs)

The expression data of RNA was acquired from The Cancer Genome Atlas (TCGA, https://cancergenome.nih.gov/). Log2 fold change > 1 and adjusted *P* < 0.05 were set as the cutoff values to screen for DEGs. Data analysis was done via R package *limma* 3.38.3.

### 2.2. Overall Survival Analysis

R packages *survival* 2.43.3 and *survminer* 0.4.3 were used to sketch the Kaplan-Meier curve and perform a log-rank test to detect the effect of the BTG2 gene on prognosis.

### 2.3. Plasmid Construction and Cell Transfection

The BTG2 sequence was synthesized and cloned into the vector pCDNA3.1+ (Invitrogen, USA), and Sanger sequencing was then carried out for verifying the final construct. Plasmids were purified with the help of DNA Midiprep Kits (Qiagen, Germany) and transfected into lung adenocarcinoma A549 cells through lipofectamine 2000 transfection reagent (Invitrogen, USA). For establishing controls, cells were transfected with blank plasmid and then screened (1.2 mg/mL G418) after 48 h transfection. Clones were selected by the limiting dilution method to obtain the stable clones with BTG2 overexpression.

### 2.4. Western Blot Analysis

The BTG2 overexpression efficiency in A549 and H1299 was detected by western blot according to standard methods. For internal control, GAPDH was used.

### 2.5. Flow Cytometry and Cell Cycle

A549 cells were grown at a concentration of 1 × 10^6^ cells per 10 cm flask, in 1640 medium with 10% FBS for 24 h, followed by harvestation for cell cycle analysis. Briefly, cells were fixed at 4°C. Prior to incubation with 50 *μ*g/mL of propidium iodide (PI), cells were collected. Finally, the stained cells were proceeded to flow cytometry analysis.

### 2.6. RNA Sequencing

Triplicate samples of vector A549 cells and BTG2 overexpressed cells were delivered to the company GENEWIZ for RNA sequencing. Using the list of DEGs identified above, GO and KEGG pathway analyses were conducted using Metascape. Metascape was also utilized for visualizing the network of protein-protein interaction (PPI). Modular analysis was carried out via Metascape.

### 2.7. Statistical Analysis

GraphPad Prism 8.3.0 was utilized for statistical calculations. Intergroup comparisons were done via an independent sample *t*-test. A *P* value of < 0.05 was deemed statistically significant.

## 3. Results

### 3.1. BTG2 is Downregulated in LUAD and Correlates with a Poor Prognosis

**Figure 1:**
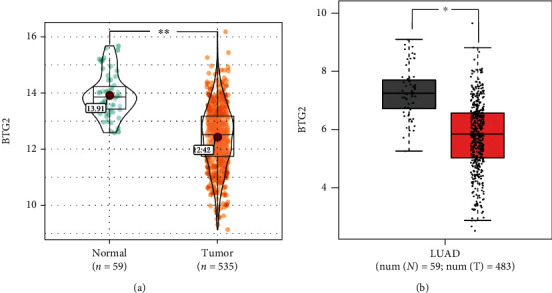
BTG2 was downregulated in LUAD tissues. BTG2 expression levels in LUAD cancer patients compared to normal samples from TCGA through R package limma 3.38.3 (a) and GEPIA online analysis (b).

**Figure 2:**
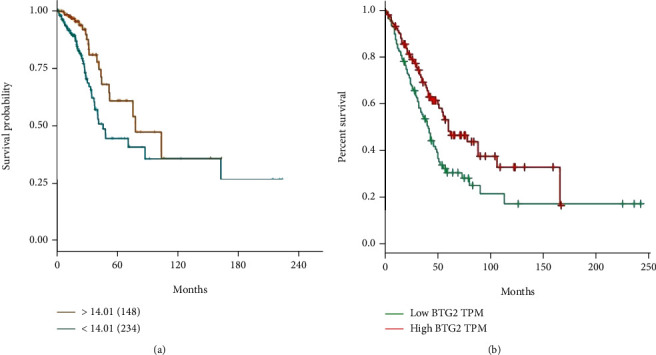
Kaplan-Meier plot showing the association between BTG2 expression and survival. (a) Analysis performed from TCGA in R package limma 3.38.3 and (b) GEPIA online database.

The mRNA expression data of 594 lung adenocarcinoma patients were downloaded from the LUAD dataset collected from TCGA. The BTG2 expression in LUAD was assessed by R package *limma* 3.38.3. Our study demonstrated that BTG2 is downregulated in LUAD than in nondiseased tissues (Figure 1(a)). The lower BTG2 levels in LUAD than the normal tissues were further validated in the Gene Expression Profiling Interactive Analysis database (GEPIA, http://gepia.cancer-pku.cn/) (Figure 1(b)). Furthermore, the association between the expression BTG2 and overall survival was assessed by the Kaplan-Meier survival curve with a log-rank comparison. LUAD patients expressing lower BTG2 showed poorer survival than those with higher BTG2 levels (Figure 2). Aforementioned results indicate that BTG2 is downregulated in LUAD, and the low expression of BTG2 predicts poor prognosis. Thus, it seems that BTG2 is a tumor suppressor in LUAD.

### 3.2. Construction of BTG2 Eukaryotic Expression Vector and A549-BTG2 Cell Clone

To generate the BTG2 eukaryotic expression vector, the BTG2 DNA fragment was amplified and digested. The 548 bp BTG2 fragment was inserted into the plasmid pDNA3.1+ resulting in the pcDNA-BTG2 vector construct. The result of Sanger sequencing confirmed that the BTG2 coding sequence was successfully cloned into pcDNA3.1 (+) vectors. Meanwhile, the Sanger recombinant plasmid sequence was proved correct (data not shown).

**Figure 3:**
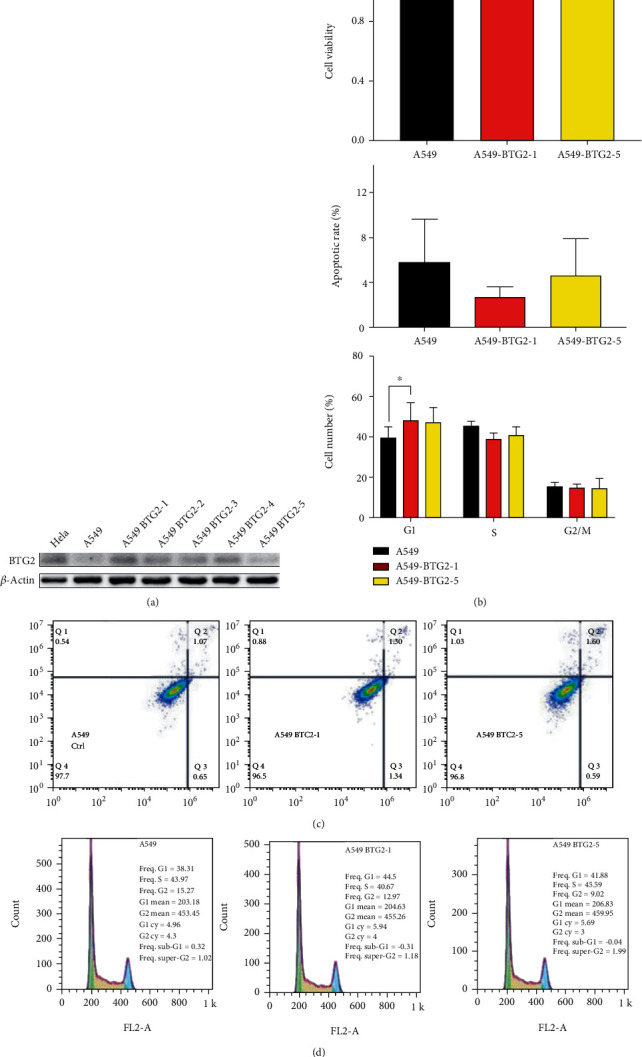
(a) The Western blot of BTG2 expression in selected clones of parental and BTG2 overexpressed cells. BTG2 expression level in HeLa cells served as a positive control and *β*-actin level as an internal control. (b) Cell growth determined by CCK-8 assays in BTG2- or vector-transfected A549 cells. (c) Representative cell cycle and flow cytometry data. (d) Cellular apoptosis was assessed via flow cytometry analysis using Annexin V-FITC and PI double staining. Data are presented as the mean ± SD and represent three independent experiments. ^∗^*P* < 0.05 by Student's *t*-test.

To explore the functional role of BTG2 at the cellular level, we established stably pcDNA-BTG2-transfected A549 cell clones in vitro. G418 was used to select the stably transfected cell clones. It turned out that 5 cell clones survived in G418. Western blotting results showed that the five cell clones express BTG2, while the cells without BTG2 transfection had no significant product. The cell clone A549-BTG2-1 expressed BTG2 and was considered the cell model for further experiments (Figure 3(a)).

### 3.3. Cell Function Experiments of A549 Cells

Cell proliferation and apoptosis analysis were studied by CCK-8 assay and flow cytometry analysis, respectively, to determine the effects of BTG2 overexpression on A549 cells. The data revealed that overexpression of BTG2 did not inhibit cell proliferation and apoptosis (Figures 3(b) and 3(c)). Later on, the effect of BTG2 overexpression was examined for the cell cycle. It revealed a significant increase in the sub-G1 phase in the BTG2 overexpressed cells compared with wild-type A549 (Figure 3(d)), which suggests that BTG2 induced cell cycle arrest. All these indicate that BTG2 induces cell cycle arrest but has no apparent effects on tumor cell growth.

### 3.4. BTG2 Downregulates the Cell Cycle-Related Genes and Activates the NF-*κ*B Pathway

To further determine the underlying mechanisms, we performed RNA sequencing of A549 cells after overexpressing BTG2 to evaluate the effects of BTG2 on the transcriptome.

All text samples were compared with the entire control sample group for obtaining differentially expressed genes (DEGs). 1040 genes were found to have significantly altered expression, including 601 upregulated and 439 downregulated genes.

**Figure 4:**
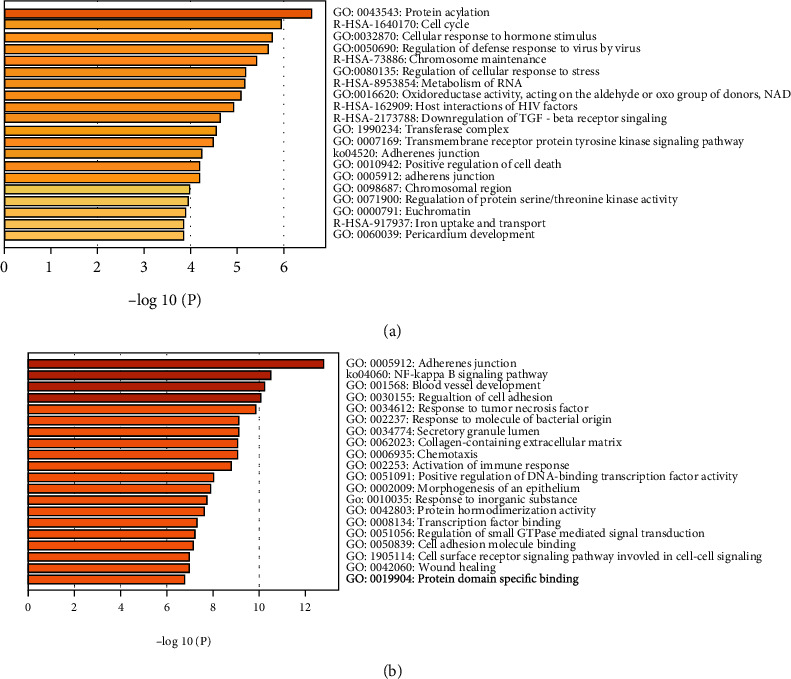
Analysis of GO and KEGG pathways of downregulated genes (a) and upregulated genes (b).

Gene Ontology (GO) and Kyoto Encyclopedia of Genes and Genomes (KEGG) analyses were carried out to evaluate if DEGs are up- or downregulated to predict mRNA functions and molecular interactions among these genes. The top 20 enriched GO and KEGG terms are shown in Figure 4.

**Figure 5:**
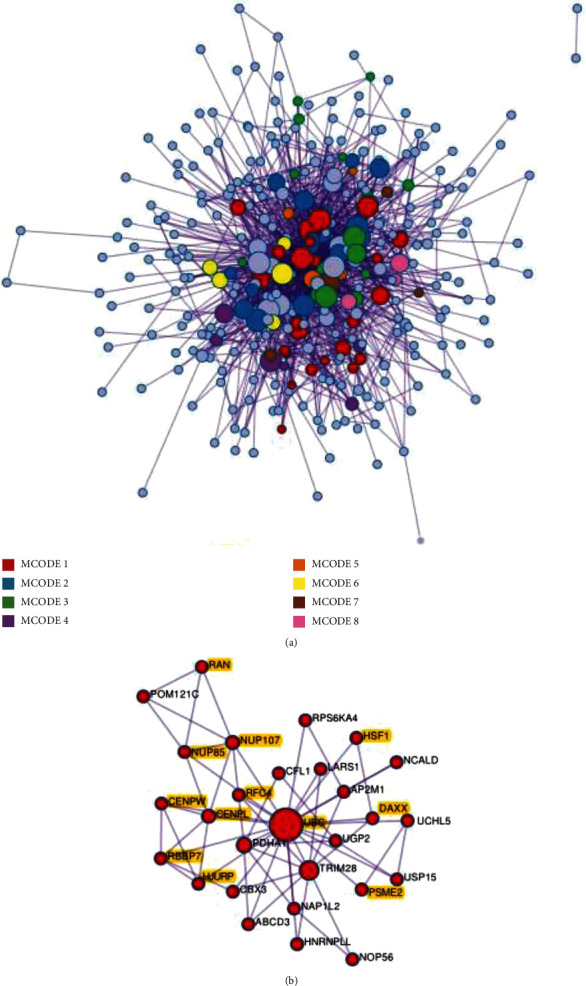
PPI network and MCODE components identified in downregulated genes. (a) PPI network of proteins. (b) MCODE1 was selected from the PPI network. Genes associated with the cell cycle are colored in yellow.

The GO analysis indicated that downexpressed genes were significantly enriched in the functional categories associated with protein acylation, cellular response to hormone stimulus, and regulation of defense response to the virus. The pathway analysis demonstrated that cell cycle, chromosome maintenance, metabolism of RNA, host interactions of HIV factors, TGF-beta receptor signaling, and iron uptake and transport were significantly enriched (Figure 4(a)). The protein-protein interaction (PPI) analysis identified in the downregulated genes is shown in Figure 5. The most significant molecular complex detection (MCODE) components were extracted from the PPI network. Twelve genes, DAXX, PSME2, RAN, RBBP7, RFC4, UBC, HJURP, NUP107, NUP85, CENPL, CENPW, and HSF1, were highlighted in MCODE 1 including cell cycle pathway (Figure 5).

**Figure 6:**
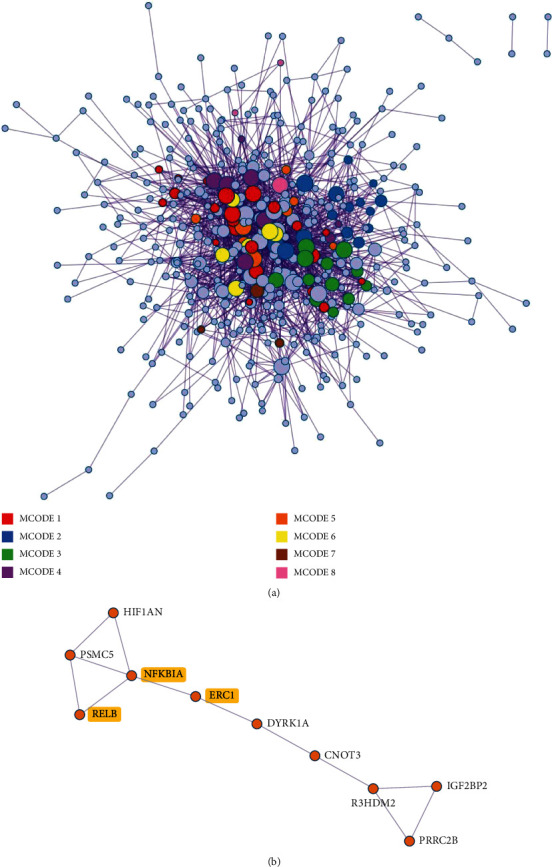
PPI network and MCODE components identified related to upregulated genes. (a) PPI network of proteins. (b) MCODE5 was selected from the PPI network. Genes involved in the signaling pathway of NF-*κ*B are colored in yellow.

The most significantly enriched GO terms of upregulated genes were a response to adhesion junction, blood vessel development, and regulation of cell adhesion, respectively. KEGG enrichment analysis revealed that highly expressed genes participated in the NF-*κ*B signaling pathway (Figure 4(b)). The PPI enrichment analysis of upregulated genes identified eight MCODE components. Three genes, NFKBIA, RELB, and ERC1, were enriched in the MCODE5 containing NF-*κ*B signaling pathway and NIK/NF-*κ*B signaling (Figure 6).

BTG2 overexpression led to significant downregulation of genes involved in cell cycle progression, consistent with findings in the flow cytometry analysis. Conversely, genes involved in the NF-*κ*B signaling pathway were significantly upregulated, indicating activation of the survival pathway upon BTG2 overexpression.

## 4. Discussion

We investigated the BTG2 function in LUAD. First, we evaluated the mRNA expression level of BTG2 and its effect on prognosis in LUAD. For further determining BTG2 gene activity, we used A549 cells with low BTG2 expression as our target cells. Then, the cell strain with BTG2 stable expression was analyzed through *in vitro* studies. Our results might provide some clues on the functional role of this gene in adenocarcinoma cancer.

Previous studies showed that BTG2 was significantly downregulated and linked with poor lung cancer patient prognosis. In line with these findings, our present data indicated that the BTG2 expression level was decreased considerably compared to normal lung tissues, and low BTG2 expression tended to have poor survival in LUAD. All these findings indicated that BTG2 is a potential lung cancer tumor suppressor.

To understand the activity of BTG2 in lung cancer, we transfected BTG2 into A549 cells. We found that BTG2 significantly induced a G1 phase cell cycle arrest, whereas cell proliferation was not influenced after BTG2 overexpression. To reveal the potential molecular mechanism, we performed RNA sequencing of BTG2 overexpressed A549 cells, and the data were then checked for DEGs. 1040 DEGs were identified; these included 601 upregulated and 439 downregulated genes. These genes may provide insight into what processes, mechanisms, and pathways are affected by BTG2.

GO enrichment analysis revealed that highly expressed genes were involved in adhesion junction response, blood vessel development, and regulation of cell adhesion. According to the KEGG pathway analysis, upregulated genes, such as NF*κ*B1, NF*κ*BIA, PLAU, PTGS2, RELB, TNFAIP3, TRAF1, ERC1, BIRC3, ATM, CD14, CD40, CXCL2, ICAM1, CXCL8, and LTB, were involved in NF-*κ*B signal pathway. NF-*κ*B transcription factors are dimers that comprise RELA, c-REL, NF-*κ*B1 (p105/p50), RELB, and NF-*κ*B2. The NF-*κ*B signaling pathway is one of the most important survival-signaling cascades after extracellular stimuli. In our study, the CCK-8 assay revealed that cell growth was not markedly decreased in overexpressed BTG2 A549 cells. On the other hand, we noted that downregulated genes affected the cell cycle pathways, which supports the outcome of FACS analysis. Such findings revealed that the NF-*κ*B activation weakens BTG2 overexpression's effect on the cell cycle. Therefore, the NF-*κ*B signaling pathway may be involved in rescuing from apoptosis in BTG2 overexpression A549 cells and helping tumor cells survive.

The TGA database assessment of the TCGA database for LUAD patients revealed that individuals with elevated levels of endogenous BTG2 expression (in tumor tissues) tend to have better clinical outcomes. Differences were also observed for the BTG2 overexpression in LUAD A549 cells. Exogenous BTG2 gene expression did not affect cell proliferation or apoptosis of A549 cells, as revealed by CCK8 and flow cytometry analyses. BTG2, as a foreign gene, randomly integrates into the host genome and will have a high expression level when inserted at a favorable position.

## 5. Conclusion

In conclusion, our results demonstrated that the overexpression of BTG2 in A549 cells might trigger survival-signaling pathways. And the p105: p50 NF-*κ*B signaling has been revealed to participate in response to BTG2 exogenous overexpression. Therefore, a potential therapeutic approach for LUAD may involve targeting BTG2 by disrupting the NF-*κ*B pathway.

## Data Availability

All the data and materials are available.
